# 4-Hydr­oxy-*N*-(2,4,6-tribromo­phen­yl)-2*H*-1,2-benzothia­zine-3-carboxamide 1,1-dioxide

**DOI:** 10.1107/S1600536809046029

**Published:** 2009-11-04

**Authors:** Muhammad Nadeem Arshad, Muhammad Zia-ur-Rehman, Islam Ullah Khan, Muhammad Shafiq

**Affiliations:** a Department of Chemistry, Government College University, Lahore 54000, Pakistan; b Applied Chemistry Research Centre, PCSIR Laboratories Complex, Ferozpure Road, Lahore 54600, Pakistan

## Abstract

In the title compound, C_15_H_19_Br_3_N_2_O_4_S, the thia­zine ring adopts a distorted half-chair conformation. The enolic H atom is involved in an intra­molecular O—H⋯O hydrogen bond, forming a six-membered ring. In the crystal, the mol­ecules are linked into a three-dimensional network through inter­molecular N—H⋯O, N—H⋯Br and O—H⋯Br hydrogen bonds.

## Related literature

For the synthesis of related mol­ecules, see: Kojić-Prodić & Rużić-Toroš (1982 ▶); Zia-ur-Rehman, Choudary & Ahmad (2005 ▶). For the applications of 1,2-benzothia­zine 1,1-dioxides and their precursor inter­mediates as non-steroidal anti-inflammatory compounds, see: Turck *et al.* (1996 ▶). For bond-length data, see: Weast *et al.* (1984 ▶).
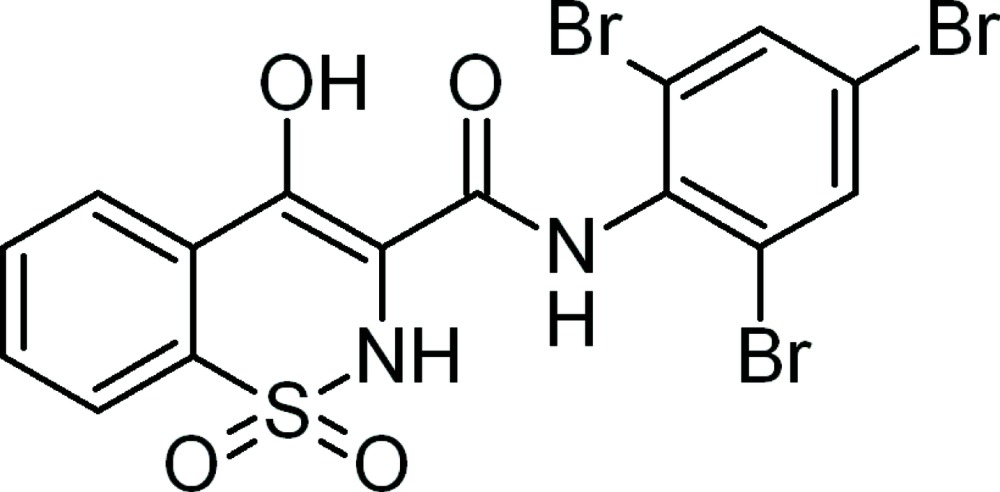


## Experimental

### 

#### Crystal data

C_15_H_9_Br_3_N_2_O_4_S
*M* *_r_* = 553.03
Triclinic, 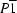

*a* = 7.5082 (4) Å
*b* = 8.7486 (6) Å
*c* = 13.0669 (9) Å
α = 83.618 (2)°
β = 86.280 (2)°
γ = 87.684 (2)°
*V* = 850.72 (9) Å^3^
*Z* = 2
Mo *K*α radiation
μ = 7.26 mm^−1^
*T* = 296 K
0.18 × 0.16 × 0.11 mm

#### Data collection

Bruker APEXII CCD area-detector diffractometer
Absorption correction: multi-scan (*SADABS*; Sheldrick, 2007 ▶) *T*_min_ = 0.355, *T*_max_ = 0.502
16515 measured reflections
3794 independent reflections
2599 reflections with *I* > 2σ(*I*)
*R*_int_ = 0.031

#### Refinement

*R*[*F* ^2^ > 2σ(*F* ^2^)] = 0.043
*wR*(*F* ^2^) = 0.116
*S* = 1.01
3794 reflections
227 parameters
H-atom parameters constrained
Δρ_max_ = 1.16 e Å^−3^
Δρ_min_ = −0.63 e Å^−3^

### 

Data collection: *APEX2* (Bruker, 2007 ▶); cell refinement: *SAINT* (Bruker, 2007 ▶); data reduction: *SAINT*; program(s) used to solve structure: *SHELXS97* (Sheldrick, 2008 ▶); program(s) used to refine structure: *SHELXL97* (Sheldrick, 2008 ▶); molecular graphics: *PLATON* (Spek, 2009 ▶) and *Mercury* (Macrae *et al.*, 2006 ▶); software used to prepare material for publication: *PLATON*.

## Supplementary Material

Crystal structure: contains datablocks I, global. DOI: 10.1107/S1600536809046029/bt5126sup1.cif

Structure factors: contains datablocks I. DOI: 10.1107/S1600536809046029/bt5126Isup2.hkl

Additional supplementary materials:  crystallographic information; 3D view; checkCIF report

## Figures and Tables

**Table 1:** Hydrogen-bond geometry (Å, °)

*D*—H⋯*A*	*D*—H	H⋯*A*	*D*⋯*A*	*D*—H⋯*A*
O4—H4⋯O3	0.82	1.83	2.561 (5)	147
N1—H1⋯O1*A* ^i^	0.86	2.29	2.966 (5)	136
N2—H2*A*⋯Br2^ii^	0.86	2.79	3.597 (4)	157
O4—H4⋯Br2^iii^	0.82	2.88	3.403 (3)	124

Symmetry codes: (i) 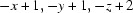; (ii) 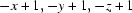; (iii) 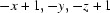.

**Fig. 1.**
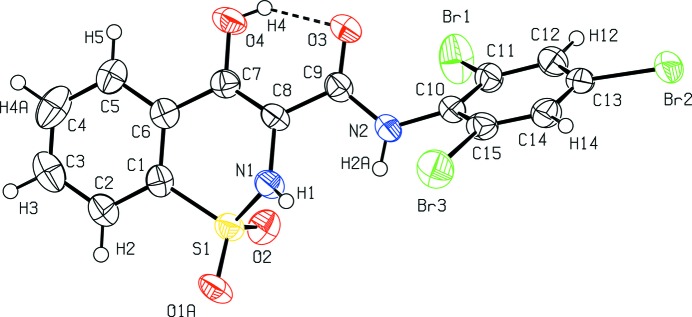
The molecular structure of the title compound, with displacement ellipsoids at the 50% probability level.

**Fig. 2.**
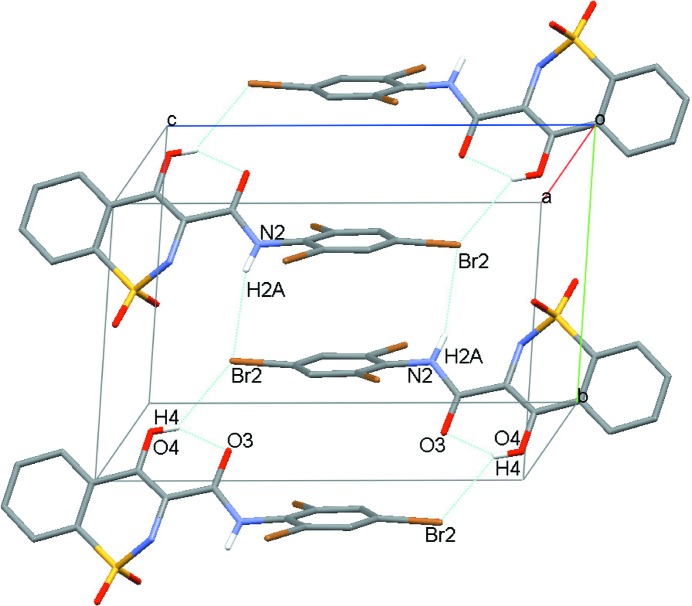
Perspective view of the three-dimensional crystal packing showing N—H···Br and O—H···Br hydrogen-bonded interactions (dashed lines). H atoms not involved in hydrogen bonding have been omitted for clarity.

**Fig. 3.**
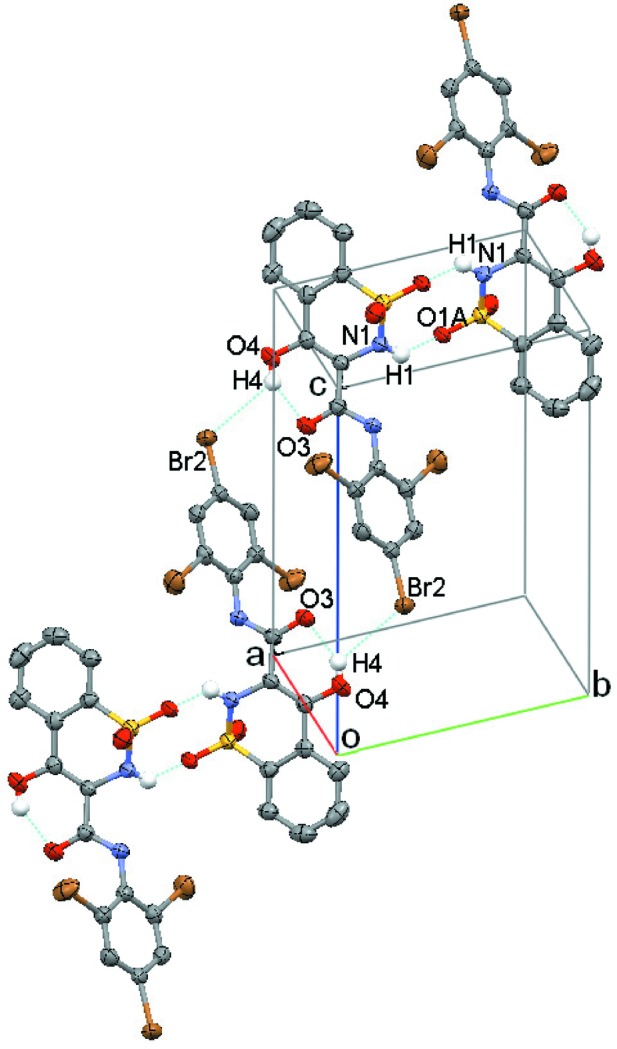
Another perspective view of the three-dimensional crystal packing showing N—H···O and O—H···Br hydrogen-bonded interactions (dashed lines). H atoms not involved in hydrogen bonding have been omitted for clarity.

## supplementary crystallographic information

### Comment

Owing to the applications of 1,2-benzothiazine 1,1-dioxides and their precursor intermediates as non-steroidal anti-inflammatory compounds (Turck *et al.*, 1996), considerable attention has been given to their synthesis. As part of a research program synthesizing 1,2-benzothiazines (Zia-ur-Rehman *et al.*, 2005), we herein report the crystal structure of the title compound, (I) (Scheme and figure 1).

The thiazine ring, involving two double bonds, exhibits a sofa conformation; with S1/C1/C6/C7 relatively planar and N1 showing significant departure from plane due to its pyramidal geometry. The enolic hydrogen on O4 is involved in intramolecular hydrogen bonding [O4—H4···O3] with the carbonyl oxygen at C9 giving rise to a six-membered hydrogen bond ring (Table 1). The C1—S1 [1.757 (5) Å] bond is shorter than a normal C—S single bond (1.81–2.55 Å) (Weast *et al.*, 1984) due to partial double bond character and is in agreement with similar molecules (Kojić-Prodić & Rużić-Toroš, 1982). Each molecule is linked to its adjacent one through intermolecular N—H···Br forming a centrosymmetric dimer which is further linked to the next *via* O—H···Br hydrogen bonds giving rise to a zigzag chain along b (Figure 2). The title molecules are also linked to each other *via* N—H···O hydrogen bonds forming dimers along b which furthr links to the adjacent dimer through O—H···Br hydrogen bonds giving rise to a zigzag chain along b (Figure 3).

### Experimental

A mixture of methyl 4-hydroxy-2*H*-1,2-benzothiazine-3-carboxylate-1,1-dioxide (2.55 g; 10.0 mmoles), 2,3-dimethyl aniline (4.947 g; 15.0 mmoles) and xylene (25.0 ml) was refluxed under nitrogen atmosphere in a Soxhlet apparatus having Linde type 4Å molecular sieves. Three fourth of the xylene was then distilled off and the remaining contents were allowed to stand overnight at room temperature. Settled solids were filtered off, washed with diethyl ether and crystallized from ethanol. Yield: 82%.

### Refinement

All hydrogen atoms were identified in the difference map and subsequently fixed in ideal positions and treated as riding on their parent atoms. In the case of the methyl and hydroxyl H atoms the torsion angles were freely refined. The following distances were used: methyl C—H = 0.98Å, aromatic C—H = 0.95Å, hydroxyl O—H = 0.84Å. U(H) was set to 1.2U_eq_ of the parent atoms or 1.5U_eq_ for methyl groups.

### Crystal data

**Table:**

C_15_H_9_Br_3_N_2_O_4_S	*Z* = 2
*M**_r_* = 553.03	*F*(000) = 532
Triclinic, *P*1	*D*_x_ = 2.159 Mg m^−^^3^
Hall symbol: -P 1	Mo *K*α radiation, λ = 0.71073 Å
*a* = 7.5082 (4) Å	Cell parameters from 5092 reflections
*b* = 8.7486 (6) Å	θ = 2.3–24.2°
*c* = 13.0669 (9) Å	µ = 7.26 mm^−^^1^
α = 83.618 (2)°	*T* = 296 K
β = 86.280 (2)°	Needle, light yellow
γ = 87.684 (2)°	0.18 × 0.16 × 0.11 mm
*V* = 850.72 (9) Å^3^	

### Data collection

**Table:**

Bruker APEXII CCD area-detector diffractometer	3794 independent reflections
Radiation source: fine-focus sealed tube	2599 reflections with *I* > 2σ(*I*)
graphite	*R*_int_ = 0.031
φ and ω scans	θ_max_ = 27.6°, θ_min_ = 1.6°
Absorption correction: multi-scan (*SADABS*; Sheldrick, 2007)	*h* = −9→9
*T*_min_ = 0.355, *T*_max_ = 0.502	*k* = −11→11
16515 measured reflections	*l* = −16→16

### Refinement

**Table:**

Refinement on *F*^2^	Primary atom site location: structure-invariant direct methods
Least-squares matrix: full	Secondary atom site location: difference Fourier map
*R*[*F*^2^ > 2σ(*F*^2^)] = 0.043	Hydrogen site location: inferred from neighbouring sites
*wR*(*F*^2^) = 0.116	H-atom parameters constrained
*S* = 1.01	*w* = 1/[σ^2^(*F*_o_^2^) + (0.049*P*)^2^ + 2.0189*P*] where *P* = (*F*_o_^2^ + 2*F*_c_^2^)/3
3794 reflections	(Δ/σ)_max_ < 0.001
227 parameters	Δρ_max_ = 1.16 e Å^−^^3^
0 restraints	Δρ_min_ = −0.63 e Å^−^^3^

### Special details

**Table:**

Geometry. All e.s.d.'s (except the e.s.d. in the dihedral angle between two l.s. planes) are estimated using the full covariance matrix. The cell e.s.d.'s are taken into account individually in the estimation of e.s.d.'s in distances, angles and torsion angles; correlations between e.s.d.'s in cell parameters are only used when they are defined by crystal symmetry. An approximate (isotropic) treatment of cell e.s.d.'s is used for estimating e.s.d.'s involving l.s. planes.
Refinement. Refinement of *F*^2^ against ALL reflections. The weighted *R*-factor *wR* and goodness of fit *S* are based on *F*^2^, conventional *R*-factors *R* are based on *F*, with *F* set to zero for negative *F*^2^. The threshold expression of *F*^2^ > σ(*F*^2^) is used only for calculating *R*-factors(gt) *etc*. and is not relevant to the choice of reflections for refinement. *R*-factors based on *F*^2^ are statistically about twice as large as those based on *F*, and *R*- factors based on ALL data will be even larger.

### Fractional atomic coordinates and isotropic or equivalent isotropic displacement parameters (Å^2^)

**Table:**

	*x*	*y*	*z*	*U*_iso_*/*U*_eq_	
Br1	0.82014 (8)	0.13368 (9)	0.54929 (5)	0.0701 (2)	
Br2	0.30934 (9)	0.34516 (6)	0.26785 (4)	0.0603 (2)	
Br3	0.17498 (9)	0.43843 (7)	0.68227 (5)	0.0629 (2)	
C1	0.8054 (6)	0.2197 (6)	1.0575 (4)	0.0373 (11)	
C2	0.8789 (7)	0.2424 (6)	1.1475 (4)	0.0462 (12)	
H2	0.9024	0.3412	1.1617	0.055*	
C3	0.9184 (8)	0.1144 (8)	1.2182 (5)	0.0591 (15)	
H3	0.9641	0.1286	1.2808	0.071*	
C4	0.8905 (7)	−0.0287 (8)	1.1956 (5)	0.0625 (17)	
H4A	0.9211	−0.1127	1.2419	0.075*	
C5	0.8152 (6)	−0.0538 (6)	1.1026 (4)	0.0454 (12)	
H5	0.7971	−0.1533	1.0878	0.055*	
C6	0.7687 (6)	0.0711 (5)	1.0339 (4)	0.0360 (10)	
C7	0.6899 (6)	0.0507 (5)	0.9372 (4)	0.0364 (10)	
C8	0.6194 (6)	0.1678 (5)	0.8750 (4)	0.0354 (10)	
C9	0.5696 (6)	0.1459 (6)	0.7707 (4)	0.0382 (11)	
C10	0.4863 (6)	0.2808 (5)	0.6055 (4)	0.0361 (10)	
C11	0.5909 (6)	0.2214 (5)	0.5271 (4)	0.0392 (11)	
C12	0.5352 (7)	0.2323 (6)	0.4267 (4)	0.0430 (12)	
H12	0.6034	0.1874	0.3753	0.052*	
C13	0.3776 (7)	0.3105 (5)	0.4054 (4)	0.0383 (11)	
C14	0.2718 (7)	0.3729 (5)	0.4799 (4)	0.0421 (12)	
H14	0.1655	0.4259	0.4638	0.050*	
C15	0.3259 (6)	0.3557 (5)	0.5797 (4)	0.0401 (11)	
N1	0.6041 (5)	0.3207 (4)	0.9051 (3)	0.0383 (9)	
H1	0.5118	0.3798	0.8931	0.046*	
N2	0.5404 (6)	0.2763 (5)	0.7070 (3)	0.0430 (10)	
H2A	0.5560	0.3627	0.7303	0.052*	
O2	0.9238 (5)	0.3836 (4)	0.8929 (3)	0.0500 (9)	
O3	0.5570 (5)	0.0166 (4)	0.7438 (3)	0.0492 (9)	
O4	0.6955 (5)	−0.0922 (4)	0.9118 (3)	0.0470 (9)	
H4	0.6471	−0.0940	0.8575	0.070*	
O1A	0.7151 (5)	0.5147 (4)	1.0094 (3)	0.0481 (9)	
S1	0.77172 (16)	0.37606 (13)	0.96331 (10)	0.0381 (3)	

### Atomic displacement parameters (Å^2^)

**Table:**

	*U*^11^	*U*^22^	*U*^33^	*U*^12^	*U*^13^	*U*^23^
Br1	0.0467 (3)	0.1064 (6)	0.0550 (4)	0.0271 (3)	−0.0078 (3)	−0.0070 (3)
Br2	0.0949 (5)	0.0455 (3)	0.0440 (3)	0.0073 (3)	−0.0311 (3)	−0.0082 (2)
Br3	0.0685 (4)	0.0635 (4)	0.0548 (4)	0.0185 (3)	0.0116 (3)	−0.0145 (3)
C1	0.032 (2)	0.046 (3)	0.034 (3)	0.0044 (19)	−0.0033 (19)	−0.005 (2)
C2	0.048 (3)	0.052 (3)	0.041 (3)	−0.002 (2)	−0.008 (2)	−0.011 (2)
C3	0.051 (3)	0.085 (5)	0.044 (3)	−0.008 (3)	−0.007 (3)	−0.018 (3)
C4	0.043 (3)	0.067 (4)	0.070 (4)	−0.005 (3)	−0.004 (3)	0.024 (3)
C5	0.038 (3)	0.050 (3)	0.047 (3)	−0.003 (2)	−0.003 (2)	0.005 (2)
C6	0.029 (2)	0.042 (3)	0.037 (3)	0.0009 (19)	0.0021 (19)	−0.006 (2)
C7	0.035 (2)	0.037 (3)	0.038 (3)	0.0014 (19)	0.002 (2)	−0.007 (2)
C8	0.036 (2)	0.037 (3)	0.035 (3)	0.0008 (19)	−0.0050 (19)	−0.013 (2)
C9	0.039 (3)	0.041 (3)	0.035 (3)	0.003 (2)	−0.004 (2)	−0.009 (2)
C10	0.043 (3)	0.034 (2)	0.033 (3)	−0.002 (2)	−0.007 (2)	−0.006 (2)
C11	0.043 (3)	0.038 (3)	0.037 (3)	0.004 (2)	−0.007 (2)	−0.005 (2)
C12	0.050 (3)	0.042 (3)	0.038 (3)	0.003 (2)	0.002 (2)	−0.010 (2)
C13	0.047 (3)	0.030 (2)	0.039 (3)	−0.001 (2)	−0.011 (2)	−0.006 (2)
C14	0.044 (3)	0.037 (3)	0.046 (3)	0.005 (2)	−0.010 (2)	−0.004 (2)
C15	0.043 (3)	0.035 (3)	0.042 (3)	0.000 (2)	0.005 (2)	−0.009 (2)
N1	0.040 (2)	0.037 (2)	0.041 (2)	0.0094 (17)	−0.0127 (17)	−0.0113 (18)
N2	0.062 (3)	0.036 (2)	0.033 (2)	0.0022 (19)	−0.0145 (19)	−0.0089 (18)
O2	0.048 (2)	0.050 (2)	0.050 (2)	0.0003 (16)	0.0008 (17)	0.0019 (17)
O3	0.067 (2)	0.041 (2)	0.042 (2)	−0.0016 (17)	−0.0091 (17)	−0.0152 (17)
O4	0.056 (2)	0.041 (2)	0.047 (2)	−0.0037 (16)	0.0011 (17)	−0.0202 (16)
O1A	0.053 (2)	0.045 (2)	0.052 (2)	0.0170 (16)	−0.0234 (17)	−0.0265 (17)
S1	0.0404 (6)	0.0357 (6)	0.0399 (7)	0.0042 (5)	−0.0088 (5)	−0.0094 (5)

### Geometric parameters (Å, °)

**Table:**

Br1—C11	1.882 (5)	C8—C9	1.471 (6)
Br2—C13	1.890 (5)	C9—O3	1.230 (6)
Br3—C15	1.886 (5)	C9—N2	1.355 (6)
C1—C2	1.368 (7)	C10—C11	1.385 (7)
C1—C6	1.411 (7)	C10—C15	1.391 (7)
C1—S1	1.757 (5)	C10—N2	1.408 (6)
C2—C3	1.405 (8)	C11—C12	1.395 (7)
C2—H2	0.9300	C12—C13	1.371 (7)
C3—C4	1.345 (9)	C12—H12	0.9300
C3—H3	0.9300	C13—C14	1.363 (7)
C4—C5	1.415 (8)	C14—C15	1.381 (7)
C4—H4A	0.9300	C14—H14	0.9300
C5—C6	1.383 (7)	N1—S1	1.628 (4)
C5—H5	0.9300	N1—H1	0.8600
C6—C7	1.461 (7)	N2—H2A	0.8600
C7—O4	1.328 (5)	O2—S1	1.418 (4)
C7—C8	1.348 (7)	O4—H4	0.8200
C8—N1	1.435 (6)	O1A—S1	1.450 (3)
			
C2—C1—C6	121.6 (5)	C15—C10—N2	119.5 (4)
C2—C1—S1	119.9 (4)	C10—C11—C12	121.3 (4)
C6—C1—S1	118.2 (4)	C10—C11—Br1	121.5 (4)
C1—C2—C3	119.1 (5)	C12—C11—Br1	117.1 (4)
C1—C2—H2	120.4	C13—C12—C11	118.6 (4)
C3—C2—H2	120.4	C13—C12—H12	120.7
C4—C3—C2	120.2 (6)	C11—C12—H12	120.7
C4—C3—H3	119.9	C14—C13—C12	122.0 (5)
C2—C3—H3	119.9	C14—C13—Br2	118.1 (4)
C3—C4—C5	121.2 (6)	C12—C13—Br2	119.8 (4)
C3—C4—H4A	119.4	C13—C14—C15	118.5 (4)
C5—C4—H4A	119.4	C13—C14—H14	120.7
C6—C5—C4	119.4 (5)	C15—C14—H14	120.7
C6—C5—H5	120.3	C14—C15—C10	122.1 (4)
C4—C5—H5	120.3	C14—C15—Br3	117.9 (4)
C5—C6—C1	118.3 (5)	C10—C15—Br3	120.0 (4)
C5—C6—C7	121.3 (5)	C8—N1—S1	116.2 (3)
C1—C6—C7	120.4 (4)	C8—N1—H1	121.9
O4—C7—C8	120.9 (4)	S1—N1—H1	121.9
O4—C7—C6	115.6 (4)	C9—N2—C10	124.8 (4)
C8—C7—C6	123.4 (4)	C9—N2—H2A	117.6
C7—C8—N1	120.9 (4)	C10—N2—H2A	117.6
C7—C8—C9	121.2 (4)	C7—O4—H4	109.5
N1—C8—C9	117.7 (4)	O2—S1—O1A	117.6 (2)
O3—C9—N2	122.8 (4)	O2—S1—N1	108.5 (2)
O3—C9—C8	121.5 (4)	O1A—S1—N1	107.9 (2)
N2—C9—C8	115.8 (4)	O2—S1—C1	108.0 (2)
C11—C10—C15	117.4 (4)	O1A—S1—C1	111.6 (2)
C11—C10—N2	123.0 (4)	N1—S1—C1	101.9 (2)
			
C6—C1—C2—C3	−0.1 (7)	C10—C11—C12—C13	3.3 (7)
S1—C1—C2—C3	−174.8 (4)	Br1—C11—C12—C13	−172.9 (4)
C1—C2—C3—C4	2.5 (8)	C11—C12—C13—C14	−2.3 (8)
C2—C3—C4—C5	−2.3 (9)	C11—C12—C13—Br2	173.8 (4)
C3—C4—C5—C6	−0.3 (8)	C12—C13—C14—C15	−0.2 (8)
C4—C5—C6—C1	2.6 (7)	Br2—C13—C14—C15	−176.4 (4)
C4—C5—C6—C7	−179.7 (5)	C13—C14—C15—C10	1.8 (7)
C2—C1—C6—C5	−2.4 (7)	C13—C14—C15—Br3	−178.5 (4)
S1—C1—C6—C5	172.3 (3)	C11—C10—C15—C14	−0.8 (7)
C2—C1—C6—C7	179.9 (4)	N2—C10—C15—C14	175.5 (4)
S1—C1—C6—C7	−5.3 (6)	C11—C10—C15—Br3	179.5 (4)
C5—C6—C7—O4	−11.4 (6)	N2—C10—C15—Br3	−4.2 (6)
C1—C6—C7—O4	166.2 (4)	C7—C8—N1—S1	40.0 (6)
C5—C6—C7—C8	170.3 (5)	C9—C8—N1—S1	−135.0 (4)
C1—C6—C7—C8	−12.1 (7)	O3—C9—N2—C10	2.5 (7)
O4—C7—C8—N1	176.5 (4)	C8—C9—N2—C10	−178.0 (4)
C6—C7—C8—N1	−5.3 (7)	C11—C10—N2—C9	−64.1 (7)
O4—C7—C8—C9	−8.7 (7)	C15—C10—N2—C9	119.8 (5)
C6—C7—C8—C9	169.5 (4)	C8—N1—S1—O2	65.2 (4)
C7—C8—C9—O3	14.5 (7)	C8—N1—S1—O1A	−166.3 (3)
N1—C8—C9—O3	−170.5 (4)	C8—N1—S1—C1	−48.6 (4)
C7—C8—C9—N2	−165.0 (4)	C2—C1—S1—O2	93.0 (4)
N1—C8—C9—N2	10.0 (6)	C6—C1—S1—O2	−81.8 (4)
C15—C10—C11—C12	−1.8 (7)	C2—C1—S1—O1A	−37.8 (5)
N2—C10—C11—C12	−178.0 (5)	C6—C1—S1—O1A	147.4 (4)
C15—C10—C11—Br1	174.3 (4)	C2—C1—S1—N1	−152.7 (4)
N2—C10—C11—Br1	−1.9 (7)	C6—C1—S1—N1	32.4 (4)

### Hydrogen-bond geometry (Å, °)

**Table:**

*D*—H···*A*	*D*—H	H···*A*	*D*···*A*	*D*—H···*A*
O4—H4···O3	0.82	1.83	2.561 (5)	147
N1—H1···O1A^i^	0.86	2.29	2.966 (5)	136
N2—H2A···Br2^ii^	0.86	2.79	3.597 (4)	157
O4—H4···Br2^iii^	0.82	2.88	3.403 (3)	124

Symmetry codes: (i) −*x*+1, −*y*+1, −*z*+2; (ii) −*x*+1, −*y*+1, −*z*+1; (iii) −*x*+1, −*y*, −*z*+1.
